# Minimally Invasive Mitral Valve Procedures: The Current State

**DOI:** 10.1155/2013/679276

**Published:** 2013-12-05

**Authors:** Bhuyan Ritwick, Krishanu Chaudhuri, Gareth Crouch, James R. M. Edwards, Michael Worthington, Robert G. Stuklis

**Affiliations:** Darcy Sutherland Cardiothoracic Surgical Unit, Level 4, East Wing, Royal Adelaide Hospital, North Terrace, Adelaide, SA 5000, Australia

## Abstract

Since its early days, cardiac surgery has typically involved large incisions with complete access to the heart and the great vessels. After the popularization of the minimally invasive techniques in general surgery, cardiac surgeons began to experiment with minimal access techniques in the early 1990s. Although the goals of minimally invasive cardiac surgery (MICS) are fairly well established as decreased pain, shorter hospital stay, accelerated recuperation, improved cosmesis, and cost effectiveness, a strict definition of minimally invasive cardiac surgery has been more elusive. Minimally invasive cardiac surgery started with mitral valve procedures and then gradually expanded towards other valve procedures, coronary artery bypass grafting, and various types of simple congenital heart procedures. In this paper, the authors attempt to focus on the evolution, techniques, results, and the future perspective of minimally invasive mitral valve surgery (MIMVS).

## 1. Introduction

Minimally invasive mitral valve surgery (MIMVS) does not refer to a single approach but rather to a collection of new techniques and operation-specific technologies. These include enhanced visualization and instrumentation systems as well as modified perfusion methods, all directed toward minimizing surgical trauma by reducing the incision size [1].

## 2. History and Evolution of MIMVS

The first successful cardiac operation was performed on September 7, 1896, in Frankfurt, Germany, by Rehn [2]. The first successful cardiac valve operation was performed in 1912 by Tuffier [3] and the first successful mitral valve (MV) operation in 1923 by Cutler and Levine [4]. In 1956, Lillehei et al. repaired multiple valvular lesions through a right thoracotomy using cardiopulmonary bypass (CPB) [5]. The subsequent years have seen a glorious phase of mitral valve surgery with full sternotomy and use of conventional cardiopulmonary bypass techniques. This phase also witnessed the development of various valvular prostheses and mitral valve repair techniques. In the 1990s, the success of laparoscopic operations in general surgery renewed an interest in minimally invasive approaches for cardiac surgery. Navia and Cosgrove [6] and Cohn et al. [7] performed the first minimally invasive valve operations (via the right parasternal and transsternal approaches). These authors have shown that small incision mitral valve surgery can be conducted safely with equivalent outcomes.

Carpentier et al. [8] in February of 1996 performed the first video-assisted mitral valve repair (MVR) through a mini thoracotomy using ventricular fibrillation. Following this the East Carolina University group performed the first video-assisted mitral valve repair through a mini thoracotomy, using video-direction, a transthoracic aortic clamp, and retrograde cardioplegia [9]. In 1998, Mohr et al. reported the Leipzig University experience using port-access technology, which was based on endoaortic balloon occlusion (EABO) rather than direct aortic clamping [10]. The next major development was the introduction of a voice-controlled robotic camera arm (AESOP 3000, Computer Motion Inc., Santa Barbara, CA, USA) which allowed precise tremor-free camera movements with less lens cleaning. This technology translated into reduced cardiopulmonary bypass (CPB) and cross-clamp (XC) times [11, 12] and enabled even smaller incisions with better valve and subvalvar visualization. The next major leap in the evolution of MIMVS was the development of robotic telemanipulation, and in 1998 Carpentier et al. [13] performed the first completely robotic mitral valve repair using the Da Vinci Surgical System (Intuitive Surgical, Inc., Sunnyvale, CA).

An important adjunct in the evolution of mini-valve surgery (mini-VS) is the parallel progress in perfusion technology [14]. First, smaller, nonkinking arterial and venous cannulae have been combined with vacuum-assisted venous drainage to allow maximal space use provided by the smaller incisions. Second, the implantation of transjugular coronary sinus catheters provides cardiac protection via retrograde cardioplegia. Third, the application of carbon dioxide (CO_2_) into the operating field limits intracardiac air (to reduce air embolism), and finally intraoperative transesophageal echocardiography allows for real-time monitoring of cardiac distention, deairing, and cannula placement [15]. Thus, MIMVS has evolved into a routinely performed operation with excellent results in many specialized centers [14, 16–18].

**Figure 1:**
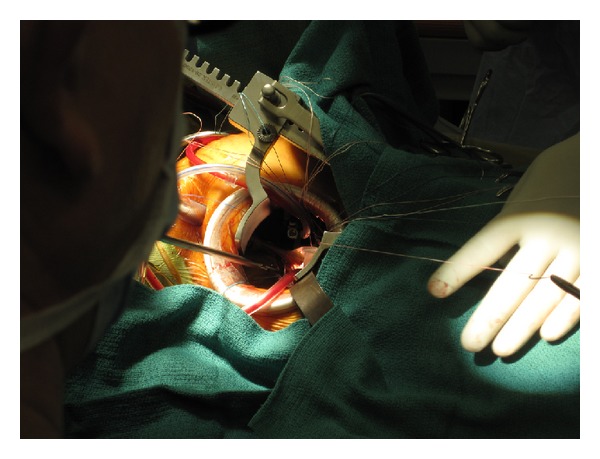
Level 2 minimally invasive approach (4–6 cm incision).

Minimally invasive valve surgery evolved through graded levels of difficulty with less exposure and to a progressive reliance on video assistance. Loulmet and Carpentier classified these levels of minimally invasive cardiac surgery as shown in Box 1 (Figure 1). Current patient selection is shown in Box 2 [19].

**Figure 2:**
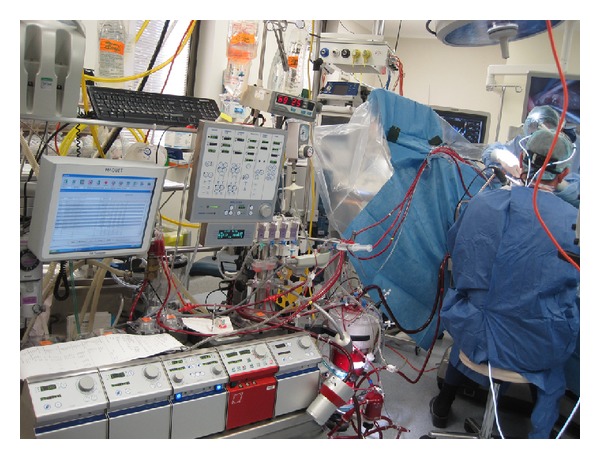
Heart Lung machine with peripheral cannulation via the femoral vessels.

**Figure 3:**
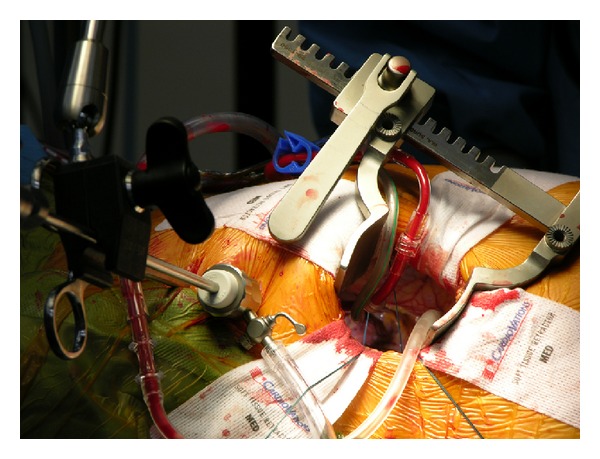
Direct transthoracic aortic clamping.

The type of the musculoskeletal incision remains central to the discussion around minimally invasive cardiac surgery. A wide variety of modified small sternal, parasternal, and minithoracotomy incisions are used to access the cardiac valves. Although many surgeons prefer the hemisternotomy approach, a right minithoracotomy yields excellent exposure for both direct vision and videoscopic mitral valve access [19]. By the mid-1900s, parasternal and transsternal approaches were being described by Navia and Cosgrove [6] and Cohn et al. [7]. Smaller incisions lateral to the sternum were created, with or without resection of the third or fourth costal cartilage. However, their disadvantages included femoral CPB cannulation, ligation of the right internal thoracic artery, occasional chest wall instability, and difficult conversion to full sternotomy. In 1997, Cohn et al. [7] presented 84 minimally invasive cases (41 aortic and 43 mitral) using a right parasternal incision and excising the third and fourth costal cartilages. Interestingly, greater patient satisfaction, a decrease in postoperative atrial fibrillation (AF), and overall lower costs were found [7]. Later, Greelish et al. [20] primarily used a lower mini-sternotomy for mini-MVS with excellent results. Chitwood et al. [21] designed a new aortic clamp that allows transthoracic aortic occlusion. Video assistance has also been used for mini-MVS through small thoracotomies [9, 16, 17]. Although there are highly encouraging results using a right thoracotomy, several disadvantages exist, including peripheral CPB cannulation, the potential need for a double-lumen endotracheal tube, and occasional difficulty with MV exposure [16]. In contrast to this, the Leipzig Group has shown excellent results with their 5-6 cm right lateral minithoracotomy under video assistance with peripheral femoral cannulation (Figure 2), direct transthoracic aortic clamping and with single endotracheal tube (Figure 3), and use of cannulation of right internal jugular vein for concomitant tricuspid valve procedures [22, 23]. Several groups strongly advocate for intra-aortic balloon occlusion for minimally invasive and robotic mitral surgery [24–27]. Most commonly these devices are introduced as retrograde through the femoral artery. The occlusive balloon is usually positioned under echocardiographic guidance just above the sinotubular junction, and balloon has the potential hazard of migration either into the arch with neurological complications or to the left ventricle with resultant ventricular dysfunction. Balloon occlusion may be advantageous compared to the transthoracic clamp method when there is limited access to the aorta. Aortic dissection is a feared complication of using the endoballoon, but experience with this technique dramatically reduces the risk of this adverse event. However, some group demonstrated increased morbidity, cost, and operative/cross-clamp time when the endoballoon technique was used for mitral valve surgery [28]. Telemanipulators, robotics that allow a hand-like mechanism to be controlled by a human operator, were first used in Paris, France, by Carpentier et al. [8] and Falk et al. [12] in Leipzig, Germany. Telemanipulator-supported operations, which involve femoral cannulation and direct or endoluminal aortic clamping, have been used and propagated by Chitwood et al. [9, 18] and others [29, 30], who claim that this technique could be safely and effectively used [7]. Other centers (Leipzig) had similar positive experiences using the telemanipulator-supported techniques in the late 1990s [31, 32]. However, they later abandoned this technique, given the lack of difference compared with their “standard” approaches. Recently in 2012, Gao et al. reported successful robotically assisted mitral valve replacement with excellent results [33].

## 3. Results

During the past 16 years, cardiac surgeons worldwide have reported their MIMVS data with promising results. The majority of these results suggest that MIMVS provide excellent, safe, and familiar exposure of the mitral valve with results comparable to those with conventional approaches. Unfortunately we lack data from large prospective randomized control series comparing the results of minimally invasive versus the conventional sternotomy technique. We therefore have to rely on retrospective analysed registry data (mostly single centre).

## 4. Mortality

Reviewing all comparative MIMVS studies evaluating mortality, no study has shown a significant difference between minimally invasive and conventional approaches [34–40]. In 2003, Greelish et al. [20] reported the first long-term results (5-year followup) of mini-VS, indicating a freedom from mitral regurgitation and reoperation >90%. In their early port access cases, Mohr et al. [23] reported a high mortality rate (9.8%) for mini-MVS, partially procedure related, with 2 of 51 patients experiencing an aortic dissection [23]. After discontinuing the port access technique and modification and simplification of the surgical procedure, the mortality decreased to an in-hospital mortality rate of 3.9% [41]. The Leipzig long-term results revealed an actuarial survival rate of 83% at 6.8 years [42]. When excluding the initial 200 patients in whom an endoclamp was used, the overall results are even more impressive [42]. In 2002, Mohr's group (Onnasch et al. [36]) reported their 5-year experience performing mini-MVS in 449 patients, with a mean survival rate of 96.3% at 2-year followup. The East Carolina University group reported a combined series with Hargrove consisting of 1178 successful video-assisted mitral valve operations between 1996 and 2008 [43]. The operative mortality rates for mitral valve repair and replacement for this two center series were 2.1% and 4.6%, respectively, but only 0.2% for isolated primary mitral valve repair. A recent meta-analysis by Modi et al. [44] identified ten papers published between 1998 and 2005 which were suitable for analysis. This study included 1358 minimally invasive patients and 1469 sternotomy patients. Although cross-clamp and cardiopulmonary bypass time were longer in the minimally invasive group, there were no difference in mortality, stroke, reoperation for bleeding, new onset atrial fibrillation, or duration of ICU stay or hospital stay [44]. In a more recent study, Stevens et al. published the results of 2,255 patients who underwent MV operations, including 1,305 with isolated MV regurgitation operations (1,054 repairs and 251 replacements) [45]. The study period was between 1992 and 2009 and surgical approaches were sternotomy in 377, video-assisted right minithoracotomy in 481, or robot-assisted in 447. Mean followup was 6.4 ± 4.5 years (maximum, 19 years) [45]. The 30-day mortality for isolated MV repair was similar for all approaches (*P* = 0.409). Fewer neurological events were observed in the videoscopic and robotic groups (*P* = 0.013). Adjusted survival was similar for all approaches (*P* = 0.357) [45]. Galloway and associates at the New York University have reported the longest outcomes for minimally invasive mitral valve surgery to date [46]. Between 1996 and 2008, they performed 1071 minimally invasive mitral valve repairs and compared their results with a cohort of 1601 conventional procedures. Almost one third of the minimally invasive repairs included an anterior leaflet procedure and all patients received an annuloplasty device [46]. They reported a perioperative mortality of 1.3% in both groups with isolated mitral valve repair and no differences in major adverse events [46]. Long-term results were equivalent to sternotomy techniques. In isolated mitral valve repair, 8-year freedom from reoperation or severe recurrent insufficiency was 93% and freedom from all the valve related complications was 90%. At the same time, they had fewer transfusions, shorter lengths of hospital stay, and fewer septic complications [46].

## 5. Neurological Events

Due to the limited access to the operative field, there is the potential for inadequate deairing of the heart leading to an increased incidence of neurological events. Mohr et al. [23] in their early series reported an 18% incidence of confusion, but were not using the CO_2_ insufflation—a technique they have since adopted. The same group after a decade observed postoperative neurological impairment in 41 of 1,339 patients (3.1%) who underwent mini-MVS, with 28 (2.1%) minor and 13 (1.0%) major events [22]. Grossi et al. [47] has recently published results of 1282 patients with an overall frequency of postoperative neurological event of 2.3% (30/1282). They also identified the high risk group for neurological event as those with peripheral vascular disease, cerebrovascular disease, dialysis, and atherosclerotic aortas [47] and also pointed out the use of retrograde arterial perfusion in diseased aortas as the most significant risk factor for the development of postoperative neurological event. In contrast to this, Gammie et al. maintained that neither retrograde arterial perfusion nor the use of end balloon were risk factors for development of postoperative neurological event [48]. This group studied 28,143 patients identified from the Society Of Thoracic Surgeons database and found a higher rate of permanent stroke, 1.87%, for the minimally invasive surgery group as opposed to 1.17% for the conventional sternotomy group) [48], and they observed a threefold higher rate of stroke in patients using fibrillatory arrest or beating heart technique without cross clamp. In their recent study, Stevens et al. have shown a reverse trend in their stroke rate (3.4%—sternotomy approach, 1.2%—videoscopic approach, and 0.7%—robotic mitral valve procedures) [49].

## 6. Bleeding Related Complications

Transfusion of allogenic red blood cells (RBCs) is recognized as a risk factor for adverse outcome after cardiac surgery [50]. Unnecessary transfusions are likely to be associated with unnecessary morbidity and additional indirect hospitalization costs.

Throughout the last decade, one of the major benefits of MIMVS has been claimed to be the less bleeding related complications and less usage of blood products [38, 51–54] as compared to the conventional sternotomy approach. Other authors have shown no difference in blood requirements in the two different groups [55]. In a recent study, Gammie et al. [48] could not show any difference in reexploration for bleeding in the MIMVS group when compared to the traditional sternotomy group but have shown a statistically significant higher use of perioperative red blood cell (52.6% for the open group and 41% for the MIMVS group) and platelet (25.3% for the open group and 15.8% for the MIMVS group) transfusion. However, when these outcomes were risk aadjusted there was no significant difference in the transfusion of either red blood cell or platelet [48]. Stevens et al. published their recent data with no difference in reexploration for bleeding in the three groups of conventional, videoscopic, and robotic mitral valve surgery (series of 2,255 patients) but with a significant difference in the requirement of blood transfusion (63%—conventional group, 43%—videoscopic, and 18%—robotic mitral valve procedures) [49].

## 7. Postoperative Atrial Fibrillation (AF)

There are conflicting data in the literature regarding the incidence of AF following MIMVS. It has been suggested that a less traumatic surgical approach would be a less potent trigger of postoperative AF. Five of six studies, however, demonstrated this not to be the case [11, 56–60], and on meta-analysis of four eligible studies, there was no significant difference between minimally invasive and sternotomy approaches (539 patients, OR 0.86, 95% CI 0.59–1.27, *P* = 0.45). More recently Gammie et al. [48], however, have shown a decreased incidence of postoperative AF (20.1% for the conventional sternotomy group and 15.9% for the less invasive group).

## 8. Septic Complications

The incidences of septic complications and wound infections are less in thoracotomy than with sternotomy. Of the three studies of mini-thoracotomy mitral valve surgery that reported wound complications compared to median sternotomy, Grossi et al. reported an incidence of 0.9% and 5.7% for mini-thoracotomy and sternotomy cases, respectively (*P* = 0.05) [61]. This increased to 1.8% and 7.7%, respectively, in elderly patients (*P* = 0.03) [37]. Santana et al. [62] recently showed a major difference of sternal wound infections and septic complications comparing minimally invasive versus sternotomy mitral valve procedures in obese patients. This group showed a 0% incidence of sternal wound infections for the minimally invasive group against a 4.1% in the sternotomy group and septic complications of 6.25% and 1.56% in the sternotomy and the minimally invasive group, respectively.

## 9. Pain and Speed of Recovery

Of all the potential benefits of MIMVS, a reduction in pain and faster return to normal activity is the most consistent finding. All four studies that measured postoperative pain levels reported less compared to sternotomy [55, 59, 62, 63], and both studies reporting time to return to normal activities noted a significant advantage for a minimally invasive approach [59, 62]. In a nonrandomized study, Cohn et al. reported equivalent pain for the first two postoperative days when a minithoracotomy approach was compared to sternotomy with a subsequent significant reduction of pain in the MI group from day 3 onwards, a difference which progressively widened with time [63]. Better stability of the bony thorax led to earlier mobilization and a faster return to activities of daily living. Glower reported that postoperative pain tended to resolve more quickly with a minimally invasive approach and that these patients returned to normal activity 5 weeks more rapidly than those having a median sternotomy (4 ± 2 weeks versus 9 ± 1 weeks, *P* = 0.01) [59]. Cohn's data is concordant with less pain in hospital and after discharge, less analgesic usage, greater patient satisfaction, and a return to normal activity 4.8 weeks ahead of sternotomy patients [62]. Walther et al. reported that 94% of his patients report no or mild postoperative pain, 99.3% feel they have an aesthetically pleasing scar, 93% would choose the same procedure again if they had to have redo surgery, and 46% are back at work within 3 weeks [64]. However, perhaps the most insightful piece of evidence for patient preference of MIMVS comes from two studies reporting that those who have had an MI approach as their second procedure all felt that their recovery was more rapid and less painful than their original sternotomy [11, 65].

## 10. Hospital Stay and Cost Savings

Vlessis and Bolling conducted a cost analysis between conventional mitral valve repair with sternotomy (ST) and MIMVS, and MIMVS was associated with a $9054 ± $3302 lower mean total hospital cost (*P* = 0.006), driven largely by a reduction in direct (*P* = 0.003) versus indirect costs (*P* = 0.06) [66]. Among the 13 billing categories, MIMVS was associated with a significant reduction in costs of cardiac imaging (*P* = 0.004), laboratory tests (*P* = 0.005), boarding and nursing (*P* = 0.001), and radiology (*P* = 0.002). More patients in the ST group required intubation for more than 72 hours (*P* = 0.019); however, there were no differences in morbidity or long-term survival (*P* = 0.334). A higher proportion of MI patients were discharged home with no nursing services (*P* = 0.018), and a higher proportion of ST patients required readmission within 1 year (*P* = 0.023). Eight of 14 studies reported a shorter hospital stay with a minimally invasive approach [7, 9, 34, 37–39, 63, 67, 68]. Only 5 studies were eligible for the meta-analysis of Modi et al. [44], and although the trend indicated this to be the case, the result was not statistically significant (350 patients, *P* = 0.07). Chitwood et al. [9], Cohn et al. [7], and Navia and Cosgrove [6] equated this trend to a 34%, 20%, and 7% cost saving, respectively. Moreover, these patients had fewer requirements for rehabilitation, a significant advantage in health care savings; 91% were discharged home compared with 67% with conventional approach [7, 67].

## 11. Operative Time

Being one of the consistent findings from various case series from the last decade, it was evident that the operative time (cardiopulmonary bypass and cross clamp time) for MIMVS is more than that of conventional surgery. There was evidence suggesting that parity can be achieved with experience while certain high volume centres report shorter operative times with MIMVS [67]. Recent study by Gammie et al. [48] with a population of 28,143 patients from the STS database also showed that the median cardiopulmonary bypass and cross-clamp times were longer in the less-invasive group compared with the conventional group (cardiopulmonary bypass time 135 versus 108 minutes, respectively; *P* < 0.0001; cross-clamp time 100 versus 80 minutes, respectively; *P* < 0.0001). The median operative time was longer (4.2 versus 3.4 hours, *P* < 0.0001) in the less-invasive group.

## 12. Intermediate and Long-Term Results

Modi et al. [44], in his meta-analysis, considered recent data from 10 cohorts with 6479 patients and found that crude unadjusted mortality rates for the entire cohort are 1.1% for mitral valve repair and 4.9% for mitral valve replacement. Galloway et al. [46] have published the longest term of followup of their MIMVS and found hospital mortality to be 2.2% for all patients (36 of 1601), 1.3% for isolated minimally invasive (9 of 712), and 1.3% (3 of 223) for isolated sternotomy mitral valve repair, as well as 3.6% (24 of 666) for valve repair plus a concomitant cardiac procedure. For isolated valve repair, 8-year freedom from reoperation was 91%  ±  2% for sternotomy and 95%  ±  1% for minimally invasive (*P* = 0.24), and 8-year freedom from reoperation or severe recurrent insufficiency was 90%  ±  2% for sternotomy and 93%  ±  1% for minimally invasive (*P* = 0.30). Eight-year freedom from all valve-related complications was 86%  ±  3% for sternotomy and 90%  ±  2% for minimally invasive (*P* = 0.14) [46].

## 13. Limitations of MIMVS

Clearly, there is a learning curve for the surgeon as well as the anesthetists, perfusionists, and nursing teams. Mohr et al. reported a high mortality (9.8%) in his early port access cases, partially procedure related with two of 51 patients suffering an aortic dissection [10]. After simplification of the surgical procedure, the mortality decreased to 3%. Vanermen et al. demonstrated that ICU and hospital stays decrease with increasing experience [24]. There are potential vascular risks with femoral cannulation, especially with the larger port access femoral cannula. Groin seromas can be problematic but are kept to a minimum by dissection of only of the anterior surface of the vessels as well as clipping lymphatics. When the pericardium is opened too posteriorly, phrenic nerve palsy has been reported and can be avoided by placing the pericardiotomy at least 3 cm anterior to it. Excess tension by pericardial retraction sutures should be avoided.

## 14. Conclusion

Cardiac valve surgery operations have historically been performed via a standard median sternotomy and CPB. With the advent of minimally invasive surgery, several new observations regarding the treatment of patients with isolated valve disease have arisen. Over the last decade there has been transformation in the way cardiac surgeons, cardiologists, and patients decide the approach to cardiac therapies. Patients now demand less-invasive procedures with equivalent safety, efficacy, and durability. Any form of new technology must provide better outcome and have better efficiency in terms of safety and durability. If scientific evidence shows that mini-VS results in lower complication rates, surgeons must be trained in these newer techniques. However, with different training backgrounds, patient populations, and surgical approaches, surgeons should use the technique that they believe will result in the best outcome and with which they feel most comfortable. The recent STS data shows that 11.3% of isolated mitral valve repairs are performed with robotic assistance [19]. Up to 20% surgeons are using some minimally invasive methods for their repairs [19].

Critically appraising the results of MIMVS has several limitations, based on the paucity of randomised controlled trials and the reliance on single centre case series or few other review papers. Furthermore, the definition of “minimally invasive” is controversial. The STS [69] defines minimally invasive surgery as any procedure not performed with a full sternotomy and CPB; however, this definition does not really fit into valve surgery.

We have attempted to review the various aspects of MIMVS, and the studies reviewed do not show a significant difference in operative mortality between minimally invasive and conventional approaches. Moreover, the long-term outcomes of these procedures appear to be as durable as the conventional approaches (with followup of up to 8 years). There has been almost no doubt that these procedures reduce the length of hospital stay and blood transfusion while at the same time being cosmetically more attractive than the conventional approach.

One of the major areas for further research is in the field of neurological outcomes as there has been conflicting data with a wide variation in the reported incidence of stroke. Most of the published series continue to implicate MIMVS done on the beating heart as increasing the risk of perioperative stroke. Further disadvantages with MIMVS are related to the use of femoral cannulation and perfusion, with groin complications (e.g., infections and arterial dissections/haematoma) accounting for morbidity unseen with conventional sternotomy.

As for the future, minimally invasive cardiac surgery is likely to become more widely adopted as growth in this niche market and cardiac surgery as a whole is often patient-driven, much in the same way that percutaneous intervention for multivessel disease has been. In essence, patients do not want a sternotomy and it is important as a surgical community that we realize this. However, despite enthusiasm, caution cannot be overemphasized as traditional cardiac operations still enjoy proven long-term success and ever-decreasing morbidity and mortality and remain our benchmark measures for comparison. To pave the path towards totally endoscopic valve surgery, surgeons, cardiologists, and engineers must focus on improving the methods of computerization of the instruments. Patient requirements, technology development, and surgeon capabilities all must be aligned to drive these needed changes. Minimally invasive valve surgery is an evolutionary process, and there must be a well-balanced alignment between the surgeons and the cardiologists to derive the maximal benefit that this technology has to offer. Traditional valve operations enjoy proven long-term success with ever-decreasing morbidity and mortality and remain the gold standard. Minimally invasive surgeries are probably not going to replace the gold standard, but they should present themselves as an alternative for treatment of mitral valve diseases with equal long-term durability.

## Figures and Tables

**Box 1:**
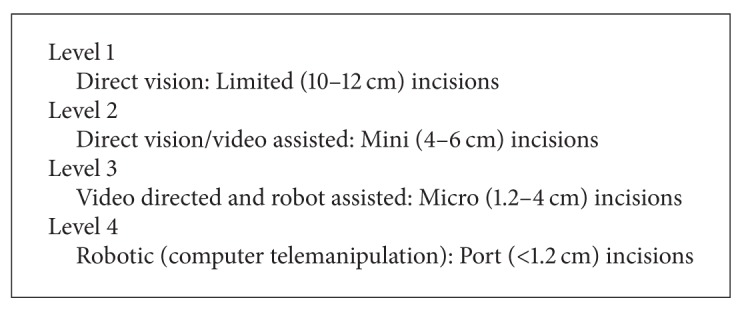
Levels of ascent in minimally invasive cardiac surgery.

**Box 2:**
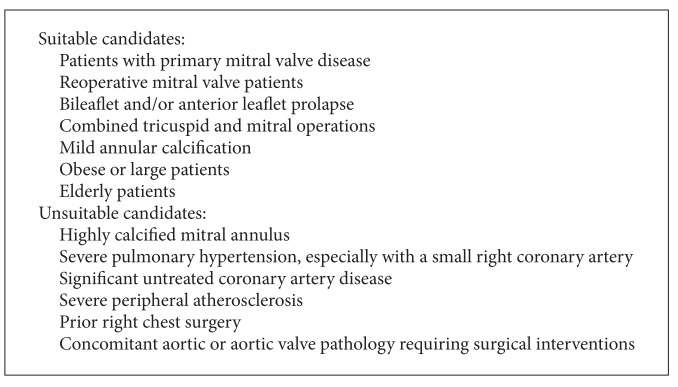
Current patient selection: videoscopic or video-assisted mitral valve surgery.
